# Human disease-causing mutations result in loss of leiomodin 2 through nonsense-mediated mRNA decay

**DOI:** 10.1371/journal.pgen.1011279

**Published:** 2024-05-15

**Authors:** Christopher T. Pappas, Rachel M. Mayfield, Ava E. Dickerson, Lei Mi-Mi, Carol C. Gregorio

**Affiliations:** 1 Department of Cellular and Molecular Medicine and Sarver Molecular Cardiovascular Research Program, University of Arizona, Tucson, Arizona, United States of America; 2 Department of Medicine and Cardiovascular Research Institute, Icahn School of Medicine at Mount Sinai, New York City, New York, United States of America; University of Michigan, UNITED STATES

## Abstract

The leiomodin (Lmod) family of actin-binding proteins play a critical role in muscle function, highlighted by the fact that mutations in all three family members (LMOD1-3) result in human myopathies. Mutations in the cardiac predominant isoform, LMOD2 lead to severe neonatal dilated cardiomyopathy. Most of the disease-causing mutations in the LMOD gene family are nonsense, or frameshift, mutations predicted to result in expression of truncated proteins. However, in nearly all cases of disease, little to no LMOD protein is expressed. We show here that nonsense-mediated mRNA decay, a cellular mechanism which eliminates mRNAs with premature termination codons, underlies loss of mutant protein from two independent LMOD2 disease-causing mutations. Furthermore, we generated steric-blocking oligonucleotides that obstruct deposition of the exon junction complex, preventing nonsense-mediated mRNA decay of mutant LMOD2 transcripts, thereby restoring mutant protein expression. Our investigation lays the initial groundwork for potential therapeutic intervention in LMOD-linked myopathies.

## Author summary

Dilated cardiomyopathy is a common type of heart disease characterized by an enlarged heart that does not contract properly. Dilated cardiomyopathy results from a variety of causes including the presence of an inherited gene mutation. Mutations in the Leiomodin 2 (*LMOD2*) gene result in a severe form of early-onset dilated cardiomyopathy that, without intervention, leads to death within weeks of birth. The majority of LMOD2 mutations discovered to date are predicted to result in the production of a truncated protein. In all cases thus far analyzed, little to no protein is made. Here we show that a cellular quality control mechanism, referred to as nonsense mediated mRNA decay, is activated to detect and destroy the instructions for making mutant LMOD2 protein, resulting in its loss. Since it was previously shown that at least one mutant LMOD2 protein is functional, we generated a treatment that blocks activation of the quality control mechanism specifically for mutant LMOD2. This treatment results in synthesis of the mutant protein and thus, could have therapeutic potential.

## Introduction

The leiomodin (LMOD) family of actin-binding proteins are critical for proper function of all types of muscle in humans. A biallelic mutation in *LMOD1*, the smooth muscle-specific *LMOD* family member, leads to megacystis microcolon intestinal hypoperistalsis syndrome, a visceral myopathy characterized by defective contractile activity of muscles that line the bladder and intestine [1]. Homozygous or compound heterozygous mutations in *LMOD3*, the isoform predominant in skeletal muscle, result in nemaline myopathy, a skeletal muscle disorder characterized by muscle weakness (there are >20 mutations reported to date) [2–6]. Additionally, disease causing mutations have been found in the cardiac-predominant isoform, *LMOD2*. We reported the first known case of a mutation in *LMOD2* (p.W398*) that results in severe neonatal dilated cardiomyopathy (DCM) [7]. Subsequently, a second case confirmed the pathogenicity of this mutation [8]. Additional mutations in LMOD2 have been discovered that also result in early-onset DCM and death [9–11]. The mutations are biallelic in all individuals, with three homozygous for the same mutation (c.273+1G>A, p.W398*, or p.L415Vfs*108) [7, 8, 10, 11] and one consisting of compound heterozygous mutations (p.R513* and p.L415Vfs*108) [9]. All data to date suggest that Lmod-linked muscle diseases have the common underlying pathophysiology of short thin filaments, reduced muscle contractility and severe muscle weakness. Most disease-causing mutations in the *LMOD* family of genes are nonsense or frameshift mutations which are predicted to result in expression of truncated proteins. However, in most cases, little to no LMOD protein is expressed [1, 2, 7]. In the case of the patient with the first described disease-causing mutation in *LMOD2* (p.W398*), LMOD2 protein was not detected in the explanted heart via western blot analysis and RT-qPCR analysis revealed a decrease in mature LMOD2 mRNA levels, but no change in pre-mRNA levels [7]. Thus, we hypothesized that nonsense-mediated mRNA decay (NMD) underlies loss of mutant protein expression. Two additional *LMOD2* disease-causing mutations (p.R513* and p.L415Vfs*108) are predicted to produce truncated proteins, however information is lacking regarding whether, and to what extent, protein is expressed [9, 10].

NMD is a surveillance system whereby the cell detects and degrades mRNAs containing premature termination codons (PTCs), however NMD also regulates the levels of certain non-mutated, functional transcripts [12, 13]. Although not completely understood, there are multiple proposed pathways by which transcripts are identified and fated for degradation [14]. One mechanism involves deposition of the multi-protein exon junction complex (EJC) upstream of exon-exon boundaries during RNA splicing [15]. The EJC remains bound to the transcript and is normally removed by the ribosome during translation. However, if a ribosome pauses at a PTC, 1) the SURF (SMG1/8/9-UPF1-eRF1-eRF3) complex of proteins can associate with the ribosome and 2) downstream EJCs remain intact. The downstream EJCs then recruit proteins (e.g., UFP3B and/or UPF2) that promote activation of the SURF complex, transforming it into the decay-inducing (DECID) complex, leading to degradation of the transcript [16].

In this study we discovered that multiple human LMOD2 mutations associated with dilated cardiomyopathy lead to a lack of LMOD2 protein expression through NMD. We also developed steric-blocking oligonucleotides to specifically inhibit NMD of LMOD2 transcripts, increasing the levels of mutant-truncated protein.

## Results

**Fig 1:**
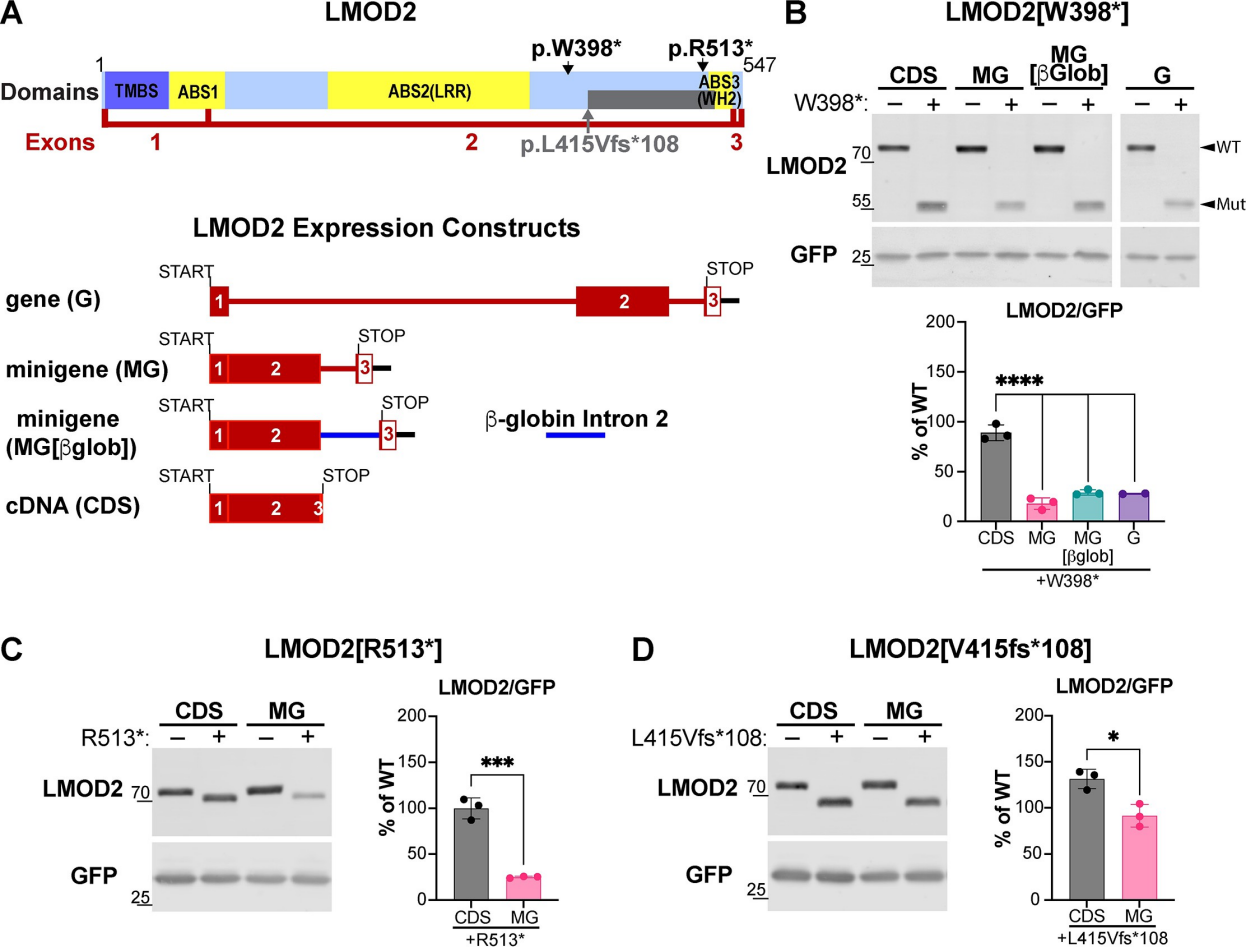
Human disease-causing mutations in *LMOD2* result in a reduction in mutant protein levels when expressed from a construct containing a downstream intron. **A**) Top, schematic of LMOD2 protein. LMOD2 contains a tropomyosin binding site (TMBS) and three actin-binding sites (ABS1,2,3). ABS2 is comprised of a leucine-rich repeat motif (LRR) and ABS3 is a WASP-homology 2, or Wiskott-Aldrich homology 2, (WH2) domain. A grey bar denotes the length of alternate amino acids that follow the L415V frame shift. p., protein. Exons are marked in red. Bottom, schematic of LMOD2 expression constructs generated for this study. Boxes represent exons and intervening lines represent introns. The translational start and stop sites are indicated. **B-D**) Western blot analysis of LMOD2 and GFP protein levels in lysate from AD-293 cells transfected with human *LMOD2* gene constructs without (–) or with (+) disease-causing mutations. **B**) LMOD2 W398*. **C**) LMOD2 R513*. **D**) LMOD2 L415Vfs*108. To control for potential transfection differences LMOD2 protein levels were normalized to co-transfected GFP. Mutant protein levels are presented as a percentage of wild type (WT) protein levels. Data are means ± SD. n = 2–3; ****P <0.0001, One-way ANOVA with Tukey’s multiple comparison test (**B**); n = 3; *P < 0.05, **P < 0.01, Student’s t-test (**C, D**).

The human *LMOD2* gene consists of 3 exons and 2 introns. In order to study the effects of disease-causing mutations on *LMOD2* mRNA and protein levels we generated human *LMOD2* gene constructs consisting of the coding sequence plus both introns (gene [G]), the coding sequence plus intron 2 only (minigene [MG]), and the coding sequence alone (CDS) (**Fig 1A**). Protein expression of constructs with and without patient mutations was analyzed in a human cell line (AD-293, which are derived from HEK293 cells) that does not express detectable levels of LMOD2 protein via western blot analysis, providing an LMOD2-free background in which to study the effect of mutations on LMOD2 protein levels. Introduction of a disease-causing mutation (W398*) into the *LMOD2* CDS construct results in a truncated protein whose expression level is slightly lower than wild type CDS (average of 89% of wild type levels), although the decrease is not statistically significant; p = 0.14, one sample t-test (**Fig 1B**). Note, the truncated protein sometimes resolves as two bands that are close in size. Conversely, the mutation results in a significant decrease in LMOD2 protein levels when introduced into constructs containing introns (MG and G) (~20–30% of wild type [WT] levels, **Fig 1B**). Protein levels are reduced to the same extent in the construct with both introns (G) and the construct without intron 1 (MG), indicating that only the presence of intron 2 is necessary for loss of mutant protein. To determine if there are specific regulatory sequences within intron 2 responsible for regulating the levels of *LMOD2*, intron 2 was replaced with the second intron of the human β-globin gene. The reduction of mutant protein levels was similar regardless of the sequence, or length, of intron 2 (**Fig 1B**).

We next tested two other *LMOD2* mutations that lead to DCM in humans. LMOD2 R513* resulted in a significant decrease in mutant protein levels only when present in the MG construct (~25% of WT levels, **Fig 1C**), while LMOD2 V415fs*108 mutant levels were only slightly lower compared to WT in the MG construct (~90% of WT levels, **Fig 1D**). However, the latter mutation resulted in an increase in protein levels when inserted into the CDS construct (~130% of WT levels, **Fig 1D**), resulting in a significant difference in the relative mutant proteins levels in the CDS vs MG constructs. These results suggest that the V415fs*108 mutation leads to increased stability of mutant protein and both mutants result in loss of protein when expressed from constructs containing introns, although the R513* mutant to a much larger degree.

**Fig 2:**
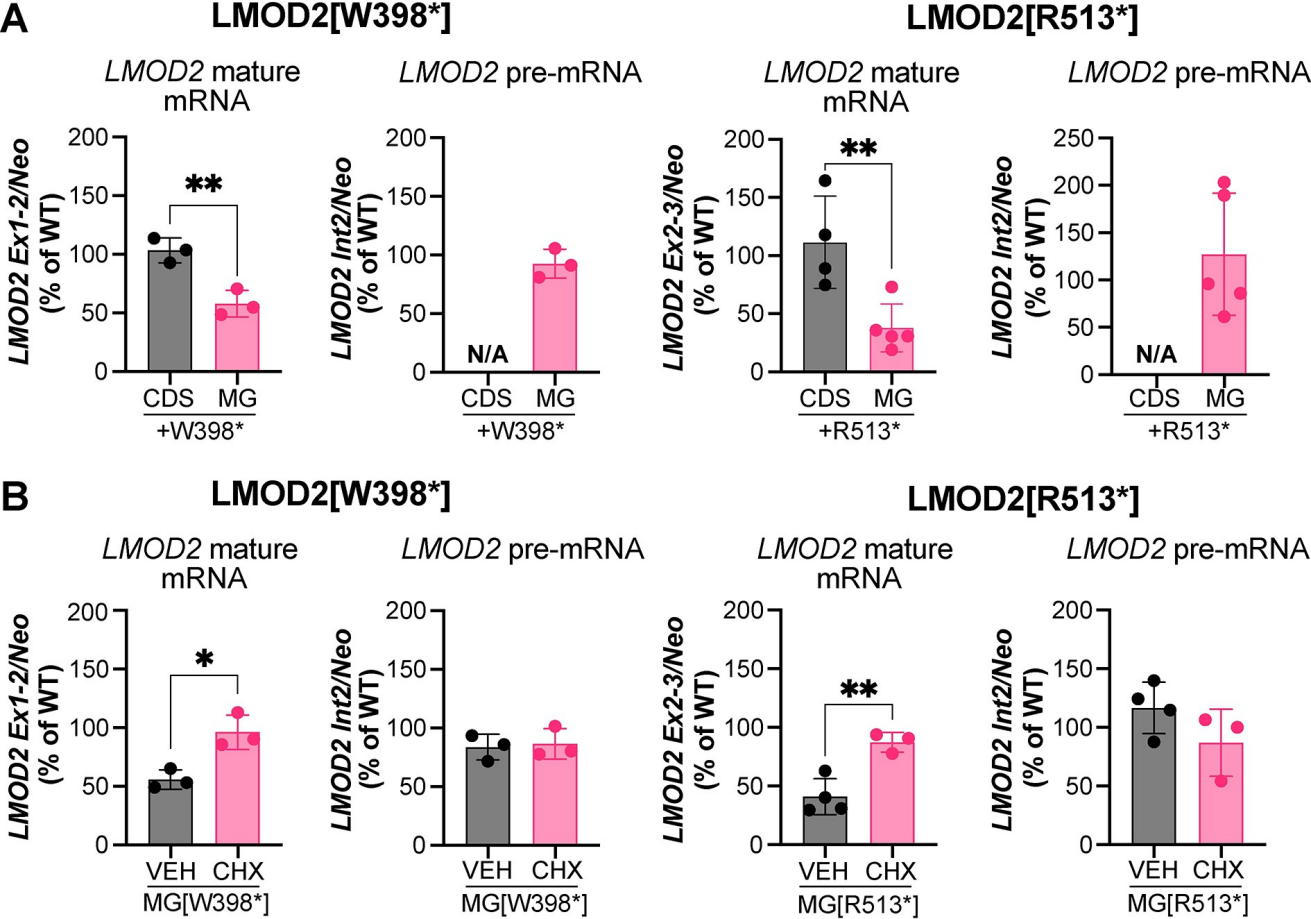
Human disease-causing mutations in *LMOD2* result in a decrease in mature, but not pre-, mRNA levels when expressed from a construct containing a downstream intron, which is ameliorated by blocking translation. **A**) RT-qPCR analysis of *LMOD2* mature mRNA and pre-mRNA levels in AD-293 cells expressing *LMOD2* coding sequence (CDS-black) or minigene constructs (MG-pink) containing the W398* (left panel) or R513* (right panel) mutations, shown as a percentage of wild type (WT) mRNA levels. **B**) RT-qPCR analysis of *LMOD2* mature mRNA and pre-mRNA levels in AD-293 cells expressing *LMOD2* minigene (MG) constructs containing the W398* (left panel) or R513* (right panel) mutations treated with vehicle alone (VEH) or 100 μM cycloheximide (CHX) for 4–5 h, shown as a percentage of wild type (WT) mRNA levels. Data are means ± SD; n = 3–5; *P < 0.05, **P < 0.01, Student’s t-test.

To determine whether the decrease in mutant LMOD2 protein levels corresponds to a reduction in *LMOD2* transcript levels we employed reverse transcriptase quantitative polymerase chain reaction (RT-qPCR). The addition of W398* and R513* mutations result in a decrease of *LMOD2* MG mature mRNA levels but have no effect on *LMOD2* CDS mature mRNA levels, when compared to their respective WT constructs (**Fig 2A**). Mutant *LMOD2* MG pre-mRNA levels are not significantly different than WT pre-mRNA levels (**Fig 2A**). This indicates that loss of *LMOD2* is occurring post-transcriptionally. Activation of nonsense-mediated mRNA decay (NMD) requires an initial round of translation, therefore blocking translation should result in the accumulation of transcripts containing PTCs, if NMD indeed contributes to loss of mature mutant LMOD2 transcript. To test for this, we treated AD-293 cells expressing *LMOD2* MGs with and without the W398* or R513* mutations with 100 μg/mL of the translation inhibitor cycloheximide (CHX) for 4–5 h. RT-qPCR analysis revealed an increase in mature mRNA levels following treatment with CHX vs vehicle (VEH) alone, while pre-mRNA levels remained unchanged (**Fig 2B**). These data are consistent with NMD as the underlying mechanism responsible for the decrease in mutant transcript levels.

**Fig 3:**
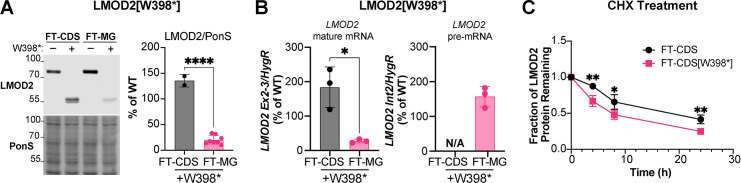
Nonsense-mediated mRNA decay is comparable in cells stably expressing of *LMOD2*[W398*] minigene constructs. LMOD2[W398*] mutant protein is slightly less stable than WT protein. **A**) Western blot analysis of LMOD2 protein levels in FT-293 cells stably expressing *LMOD2* gene constructs without (–) or with (+) the W398* mutation, presented as a percentage of wild type (WT) protein levels **B**) RT-qPCR analysis of *LMOD2* mature mRNA (left) and pre-mRNA (right) levels in FT-293 cells stably expressing *LMOD2* coding sequence (CDS-black) or minigene constructs (MG-pink) containing the W398* mutation, shown as a percentage of wild type (WT) mRNA levels. **C)** Cycloheximide (CHX) chase experiment. LMOD2 protein levels in FT-293 cells stably expressing the LMOD2 CDS construct without (black) or with (pink) the W398* mutation treated with 300 μM CHX for 4, 8 or 24 h. Data are means ± SD; n = 2–8 (**A**), 3 (**B**), and 4 (**C**); *P < 0.05, **P < 0.01, ****P < 0.0001, Student’s t-test.

It has been reported that, in some instances, the efficiency of NMD is decreased for transcripts expressed via transient transfection compared to the same construct stably expressed [17]. Therefore, to determine if our initial results are an underestimate of the degree to which mutant *LMOD2* mRNA is degraded, we generated cell lines stably expressing *LMOD2* CDS and MG constructs with and without the W398* mutation (Flp-In TREx-HEK293 [FT-293] cells). Western blot analysis indicated a nearly equivalent reduction in LMOD2[W398*] protein levels as that found following transient transfection (**Fig 3A**). However, the stably expressing cells displayed a larger decrease in mutant *LMOD2* MG mature mRNA levels compared to that observed with transient transfection (**Fig 3B**), while the pre-mRNA levels were not changed (**Fig 3B**). To determine if the decrease in the levels of mutant LMOD2 protein is solely due to loss of transcript by NMD or if the stability of LMOD2 protein is affected by the W398* mutation, a cycloheximide (CHX) chase experiment was conducted. FT-293 cells stably expressing the *LMOD2* CDS, with or without the W398* mutation were treated with CHX and levels of LMOD2 protein monitored over time via western blot analysis. The CDS constructs were used to avoid loss of mutant protein due to NMD. There was a reduction (~25–40%) in LMOD2[W398*] protein at all timepoints measured compared to wild type LMOD2 (**Fig 3C**), suggesting that, in addition to NMD, a decrease in stability contributes to loss of mutant protein.

**Fig 4:**
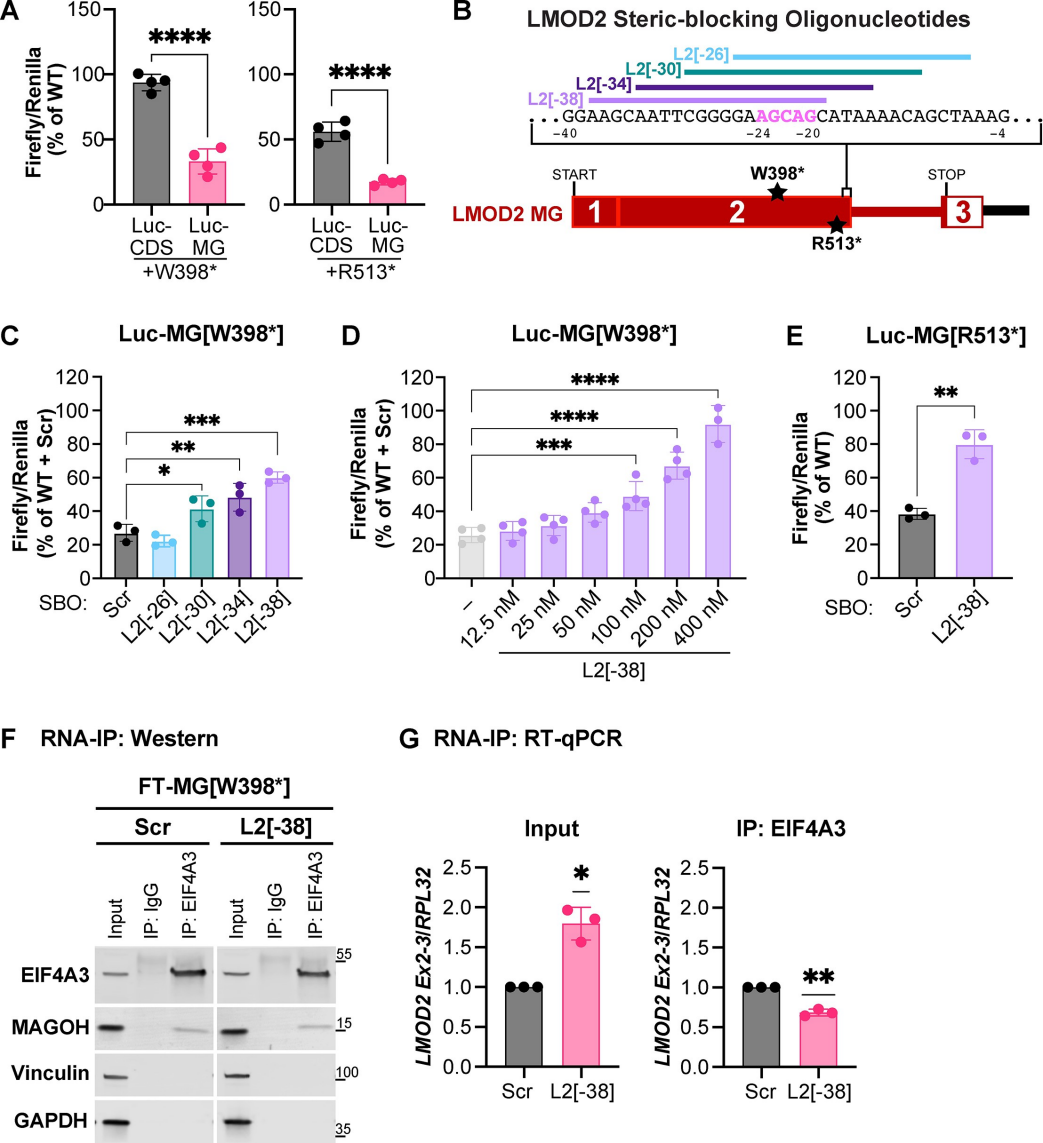
Nonsense-mediated mRNA decay of the *LMOD2*[W398*] minigene construct is inhibited by treatment with steric-blocking oligonucleotides that hinders deposition of the EJC. **A**) Normalized firefly luciferase activity for Luc-*LMOD2* CDS and MG constructs with and without the W398* (left) or R513* (right) mutations presented as a percentage of wild type (WT) protein levels. Data are means ± SD. n = 4; ****P <0.0001, Student’s t-test. **B**) Schematic of *LMOD2*-MG construct with boxes representing exons and intervening lines representing introns. The locations of the W398* and R513* mutations, SBOs and the putative EJC binding site (pink) upstream of the junction between exons 2 and 3 are indicated. **C**) Normalized firefly luciferase activity for Luc-*LMOD2* MG[W398*] treated with 100 nM of the steric-blocking oligonucleotides (SBOs) indicated. Data are presented as the mean percentage of Luc-*LMOD2* MG (WT) + Scr SBO activity ± SD. n = 3; *P <0.05; **P <0.01; ***P <0.001, One-way ANOVA with Dunnett’s multiple comparison test. **D**) Normalized firefly luciferase activity for Luc-*LMOD2* MG[W398*] treated with various concentrations of the L2[-38] SBO. Data are presented as the mean percentage of Luc-*LMOD2* MG + 100 nM scrambled SBO ± SD. n = 4; ***P <0.001,****P <0.0001, One-way ANOVA with Dunnett’s multiple comparison test. **E**) Normalized firefly luciferase activity for Luc-*LMOD2* MG[R513*] treated with 400 nM of the steric-blocking oligonucleotides (SBOs) indicated. Data are presented as the mean percentage of Luc-*LMOD2* MG (WT) activity ± SD. n = 3; **P <0.01, Student’s t-test. **F**) Representative western blot of input or immunoprecipitate probed for components of the EJC (EIF4A3 and MAGOH) or control proteins (vinculin and GAPDH). **G**) RT-qPCR analysis of *LMOD2* mature mRNA levels in input lysate (left panel) or the immunoprecipitate (right panel) from cells treated with 100 nM scrambled (black) or L2[-38] (pink) SBOs. Data are means ± SD. n = 3; *P <0.05, **P <0.01, comparison with a hypothetical mean of 1.0, one sample t-test.

We next set out to block NMD in an attempt to recover mutant LMOD2 protein. In order to more efficiently screen for protein recovery, we utilized a Dual-Glo Luciferase Assay System. The *LMOD2* CDS and MG constructs (with and without the W398* mutation) were fused to the C-terminus of firefly luciferase (Luc-CDS and Luc-MG). *Renilla* luciferase is also expressed from the same plasmid under control of a separate promoter in order to control for transfection efficiency and cell death. AD-293 cells were transfected with Luc-CDS and Luc-MG constructs and the ratio of firefly:*Renilla* luminescence determined. The W398* mutation results in a decrease in luciferase activity when inserted into the Luc-MG construct (~70%), but very little change when present in the Luc-CDS construct (**Fig 4A**). The 513* mutation results in a decrease in luciferase activity when inserted into both the Luc-MG and Luc-CDS constructs, but more so in the MG construct (~80% vs. 40%, respectively) (**Fig 4A**). Thus, the luciferase assay provides a system to easily measure LMOD2 protein loss due to NMD. In order to inhibit NMD specifically for *LMOD2* we set out to block deposition of the exon junction complex (EJC) only on *LMOD2* mRNA. Canonical EJC binding sites are located 20–24 base pairs upstream of the exon-exon junction, and binding is seemingly independent of sequence identity at that location [15]. Steric-blocking oligonucleotides (SBOs) that target the EJC binding site have been successfully used to inhibit NMD in the past [18, 19]. We designed four overlapping 20-22-mer SBOs (each offset by 4 nucleotides) uniformly modified with phosphorothioate backbones and 2’-O-(2-methoxyethyl) (2’-O-MOE) sugars that span the putative EJC binding site between exons 2 and 3 of *LMOD2*; as a control, a scrambled sequence was also generated (**Fig 4B**). The modifications provide the SBOs with increased nuclease resistance and improved binding affinities, as well as prevent knockdown of the targeted transcript via RNaseH-mediated cleavage of the SBO-RNA duplex [20]. 100 nM of each SBO was cotransfected with Luc-MG[W398*] into AD-293 cells. Treatment with three of the SBOs (L2[-30], L2[-34], and L2[-38]) resulted in a significant increase in normalized firefly luciferase activity (**Fig 4C**). The most effective SBO (L2[-38]) displays dose-dependent activity with a nearly full recovery of mutant protein levels to WT levels at the highest concentration tested (**Fig 4D**). Western blot and RT-qPCR analysis confirmed that the SBOs result in an increase in mutant protein and mature mRNA levels, respectively (**S1 Fig**). Transfection of L2[-38] SBO also increases expression of LMOD2[R513*] mutant protein (**Fig 4E**). Thus, we have identified SBOs that are effective at recovering mutant LMOD2 protein expression.

To assess whether the L2[-38] SBO does indeed block deposition of the EJC on *LMOD2* mRNA we used RNA-IP (RIP). A core component of the EJC (EIF4A3) was immunoprecipitated from FT-293 cells stably expressing *LMOD2* MG[W398*] (**Fig 4F**). Probing for another component of the EJC, MAGOH, indicated that the greater complex was pulled down. RNA associated with the complex (or in the input lysate) was isolated, cDNA generated and RT-qPCR used to quantify transcript levels. Analysis of RIP input lysate revealed an increase in *LMOD2* MG[W398*] transcript levels following treatment with SBO L2[-38] compared to treatment with a scrambled SBO, consistent with inhibition of NMD (**Fig 4G**). However, the amount of LMOD2 MG[W398*] transcript associated with the EJC is significantly decreased following treatment with SBO L2[-38] compared to a scrambled SBO (**Fig 4G**), indicating the L2[-38] SBO interferes with deposition of the EJC on *LMOD2* mRNA.

## Discussion

In this study we developed an experimental system to directly test the effect of human *LMOD2* disease causing mutations on the expression and stability of LMOD2 protein. To date, four distinct LMOD2 mutations have been discovered in individuals from five separate families that all present with early onset dilated cardiomyopathy [7–11]. By analyzing explanted hearts we previously found that two of the mutations (p.W398* and c.273+1G>A*) result in a lack of full-length or mutant protein expression [7,11]. Here we further show the p.W398* mutation, and an additional unstudied mutation (p.R513*), result in very little mutant LMOD2 protein expression when expressed from gene constructs in a human cell line, while another unstudied mutation (p.V415fs*108) does not significantly decrease mutant protein levels. Interestingly, a patient homozygous for the latter mutation displayed the latest onset of disease to date, presenting at 9 months of age [10], revealing a potential correlation between mutant LMOD2 protein levels and severity of DCM. While the L415Vfs*108 mutation did not result in a significant decrease in mutant protein when expressed from the minigene construct (~90% of wild type levels), when expressed from the CDS construct the mutation results in an increase in protein levels (~130% of wild type) suggesting that the mutant protein, which contains 108 random residues C-terminal to valine 415, is more stable than wild type LMOD2.

Multiple lines of evidence point to nonsense-mediated mRNA decay (NMD) as the underlying cause of loss of LMOD2 protein due to the W398* and R513* mutations. First, the mutations only result in a large decrease in protein levels when expressed from constructs containing an intron, indicating that significant loss of mutant protein is not due to a defect at the level of the protein. Second, mature mutant mRNA levels are decreased, while pre-mRNA levels are unaffected, indicating that the mutation acts post-transcriptionally. Third, mature mutant mRNA levels increased upon short-term inhibition of protein synthesis with cycloheximide, which is consistent with the requirement of translation to initiate NMD. Fourth, inhibition of a known mechanism through which NMD functions (the presence of an exon junction complex downstream of a premature termination codon) partially recovers mutant protein levels.

We previously found that introduction of GFP-Lmod2 into *Lmod2* KO mice just after birth prevents onset of DCM, with the mice living to adulthood [21]. We also determined that a surprisingly small amount of Lmod2 (~15% of endogenous levels) is required to maintain function [22]. Furthermore, introduction of mouse GFP-Lmod2 with a mutation (W405*) homologous to the W398* mutation found in the first patient identified (generated from cDNA so it is not susceptible to degradation due to NMD*)* partially restores cardiac morphology and function in *Lmod2* KO mice [7]. Therefore, recovery of mutant protein expression, even at low levels, could potentially improve cardiac function in some cases of DCM due to mutations in *LMOD2*.

Various strategies have been utilized to inhibit NMD including RNA interference to knock down the expression of NMD factors [23], the use of small molecule inhibitors to directly disrupt the function of NMD factors [24, 25] or indirectly affect cellular function [26, 27] and steric-blocking oligonucleotides to block the binding of the EJC [18, 19]. Since NMD is known to regulate many endogenous transcripts, the therapeutic potential of general inhibition of NMD may be limited. Thus, we chose to employ steric-blocking oligonucleotides (SBOs) to take advantage of the specificity of Watson-Crick base pairing with the goal of only targeting the LMOD2 transcript by blocking a single EJC binding site downstream of the PTC. Three of the four SBOs (L2[–30], L2[–34], and L2[–38] effectively increased mutant LMOD2 protein expression. One SBO did not recover mutant LMOD2 protein or mature mRNA levels and resulted in an apparent increase in *LMOD2* pre-mRNA levels. The reason for the increase in pre-mRNA levels is not clear, but could result from altered pre-mRNA processing and/or degradation [28].

All of the *LMOD2* disease-causing mutations (should they be expressed) result in proteins missing varying degrees of their C-termini, which includes one of the three known actin-binding sites of LMOD2. Thus, while recovery of mutant protein expression, due to successful inhibition of NMD, is predicted to improve cardiac function and could potentially provide the patient with additional time to receive other therapies or a cardiac transplant, it is not expected to provide a cure since it is likely that fully-functional protein is not made. Supporting this conclusion, transgenic mice expressing the homologous mutation (p.W405*) to that first discovered to cause disease in humans (p.W398*) live significantly longer than *Lmod2* knockout mice, but display dilated cardiomyopathy and die prematurely as adults (Iwanski et al., in preparation). Alternatively, combination of SBO treatment with drugs that promote translational readthrough of premature termination codons could prove therapeutically synergistic. Greater than 50 compounds that promote stop-readthrough have been discovered and are in various stages of therapeutic development [29]. However, degradation of mutant transcripts containing premature termination codons by NMD could limit the effectiveness of stop-readthrough drugs. Thus, treatment with an SBO that inhibits NMD in combination with a drug that promotes translation of the full-length protein could result in rescue of cardiac function in patients with certain mutations in *LMOD2*.

This study was performed in a human cell line that does not express detectable levels of LMOD2 to facilitate analysis of LMOD2 gene constructs containing patient mutations. Thus, further research is needed to determine the extent of NMD, and the effectiveness of steric-blocking oligonucleotides, in patient-derived cardiomyocytes.

## Materials and methods

### Human embryonic kidney 293 (HEK293) cell lines/Transfections

AD-293 cells (Agilent, 240085), a derivative of HEK293 cells, were cultured in DMEM (Gibco, 11995–065) supplemented with 8% fetal bovine serum (Omega Scientific, FB-02), MEM non-essential amino acids (Gibco, 11140–050) and Pen-Strep (Gibco, 15140–122). Cells were transfected using Lipofectamine 3000 Transfection Reagent with a lipid to DNA ratio of 2:1 according to the manufacturer’s instructions (Invitrogen, L3000015). Cells were collected 48–72 hours after transfection. To generate isogenic stable HEK293 cell lines expressing LMOD2 gene constructs the Flp-In system was used according to the manufacturer’s instructions (ThermoFisher Scientific, K601002). Briefly, a host TREx-293 cell line was purchased, which contains a Flp Recombination Target (FRT) site at a single locus in the genome (ThermoFisher Scientific, R71007). Co-transfection of an expression vector (pcDNA5/FT, that contains an LMOD2 gene construct under control of a CMV promoter, an FRT site, and a hygromycin resistance gene) with a plasmid expressing Flp recombinase (pOG44) results in homologous recombination between the Flp sites and integration of the LMOD2 construct into a single site within the genome. “Polyclonal” selection of stably expressing cell lines (named Flp-In TREx-293 [FT-293]) was completed via hygromycin selection. The expression plasmid used (pcDNA5/FT) results in constitutive LMOD2 expression.

### Cycloheximide (CHX) treatment

For experiments to inhibit NMD, AD-293 cells were plated in 35 mm culture dishes, transfected the next day (at 50–80% confluency) with *LMOD2* gene constructs, and, following 2 days of expression, treated for 4-5h with 100 μg/mL of CHX resuspended in EtOH (Sigma, C7698). For CHX chase experiments, FT-293 cells were plated at 1x10^6^ cells/35 mm culture dish, and after 2 days treated with 300 μM CHX in DMSO (Sigma, 508739) for 0, 4, 8 and 24h.

### Lmod2 gene constructs

*LMOD2* and Hemaglobin subunit beta (*HBB*) gene sequences (corresponding to the NCBI human reference sequences GRCh38.14; chr7:g.123655866 – g.123664290 and chr11:complement, g.522546 – g.5227071, respectively) were cloned from human genomic DNA and the *LMOD2* coding sequence synthesized according to NCBI reference sequence NM_207163.2 (Genscript). To generate minigene (MG) constructs, fragments subcloned from the gene and cDNA sequences were combined by overlap extension PCR. Gene, minigene and cDNA sequences were inserted into pcDNA3.1(-) or pcDNA5/FRT, placing them under control of a CMV promoter. Note, the constructs do not contain the 5’ UTR of *LMOD2*. *LMOD2* disease-causing mutations were added to constructs via site-directed mutagenesis according to the protocol in [30]. Relevant sequence information is in the supplementary information (**S1 File**).

### Steric-blocking oligonucleotides

20–22 mer oligonucleotides uniformly modified with a phosphorothioate backbone and 2’-O-methoxy-ethyl (2’-MOE) bases were synthesized by Integrated DNA Technologies. The sequences, which are named according to their location upstream of the junction between exons 2 and 3 of *LMOD2*, are in the supplementary information (**S1 File**).

### Western blot analysis

Cells were washed twice with PBS and collected with a cell lifter in ice-cold lysis buffer (150 mM NaCl, 1.5 mM MgCl2, 1 mM EGTA, 10 mM sodium pyrophosphate, 10 mM sodium fluoride, 0.1 mM sodium deoxycholate, 1% Triton X-100, 1% SDS, 10% (vol/vol) glycerol, 25 mM HEPES, pH 7.4, plus 1X Halt Protease Inhibitor Cocktail [ThermoFisher Scientific, 78439]) and sonicated 4 × 10 sec on power 2. Samples were spun down for 15 min at 16,000 × g at 4°C, flash frozen and stored at -80°C until processing. Total lysate protein concentration was normalized by BCA assay (ThermoFisher Scientific, 23225) and samples incubated in 1X Laemmli sample buffer at 100°C for 10 min. 20–30 μg of total protein was resolved on a 10% SDS gel, transferred to PDVF, and the membrane stained with Ponceau S. The membrane was then scanned and allowed to dry. For probing, the membrane was blocked with 5% (wt/vol) nonfat dried milk/TBS for 1 h at room temperature followed by incubation with primary antibodies in 1% BSA (wt/vol)/TBST overnight at 4°C. Primary antibodies included: mouse monoclonal anti-GFP (0.2 μg/ml; B-2, Santa Cruz Biotechnology, sc-9996), rabbit polyclonal anti-Lmod2 (0.1 μg/ml; E13, Santa Cruz Biotechnology, sc135491); rabbit polyclonal anti-EIF4A3 (0.2 μg/ml; Proteintech, 17504-1-AP); mouse monoclonal anti-MAGOH (0.4 μg/ml; F-6, Santa Cruz Biotechnology, sc-271365); mouse monoclonal anti-vinculin (1:6000; hVIN-1, Sigma, V9131) and mouse monoclonal anti-GAPDH (1 μg/ml; Proteintech, 60004–1). The membranes were then washed 5 x 5 min in TBST and incubated with Alexa Fluor 680 or Alexa Fluor 790 AffiniPure donkey anti-rabbit or anti-mouse IgG (1:40,000; Jackson ImmunoResearch) diluted in 5% milk/TBST for 1 h at room temperature. Following 5 x 5 min washes in TBST, blots were imaged and analyzed using a LI-COR Odyssey CLx imaging system (LI-COR).

### Reverse transcription quantitative polymerase chain reaction

RNA was extracted from HEK293 cells using a Quick-RNA Miniprep Kit, including an on-column DNase digestion, according to the manufacturer’s instructions (Zymo Research). cDNA was synthesized from 500 ng of total RNA using RevertAid RT or TaqMan Reverse Transcription Kits (ThermoFisher Scientific, K1691 or N8080234) with oligo deoxythymidine (dT) or random hexamer primers. Five microliters of template cDNA (diluted 1:25) was used in a PCR with Maxima SYBR Green qPCR master mix (ThermoFisher Scientific, K0252) on a LightCycler 480 (Roche). To determine relative gene expression, the ΔΔ*C*t method was used. Each cycle threshold value (Ct) was normalized to that of a reference gene (ΔCt) and then compared with the ΔCt of a control group (typically wild type *LMOD2* CDS or MG) (ΔΔCt). The mean 2^-ΔΔCt for each group was then calculated. The neomycin resistance (Neo) gene, which is expressed from the same plasmid as the gene constructs (pcDNA3.1[–]) was used as the reference gene for transient transfections. The hygromycin resistance (Hyg) gene, which was inserted into the genome along with the *LMOD2* constructs, was used as the reference gene for the stable expression cell lines. Ribosomal protein L32 (*RPL32*) was used as the reference gene for the RNA-IP experiments since it has multiple exons and introns and is therefore predicted to associate with the exon junction complex. Primer efficiencies were determined from standard curves generated using serial dilutions of cDNA. To assess mature *LMOD2* mRNA levels, primers spanning introns were used in conjunction with cDNA amplified with an oligo dT primer. To determine *LMOD2* pre-mRNA levels, primers within intron 2 were used in conjunction with cDNA amplified with a random hexamer primer. Primer sequences are in the supplementary information (**S1 File**).

### Luciferase assays

A pmirGLO Dual-Luciferase miRNA Target Expression Vector (Promega, E1330) was modified to remove the stop codon from firefly luciferase. *LMOD2* CDS and MG constructs (with and without disease-causing mutations) were inserted in-frame downstream of firefly luciferase to generate fusion proteins. AD-293 cells were plated in 24-well culture dishes and transfected the next day (at 50–80% confluent) with luciferase-*LMOD2* fusion constructs and/or SBOs using Lipofectamine 3000 Transfection Reagent with a lipid to DNA ratio of 2:1 according to the manufacturer’s instructions (Invitrogen, L3000015). Two days later the cells were dissociated with a small volume of 0.25% trypsin (Gibco, 25200–056) and quenched with media in a final volume of 100 μL. 75 μL of cells were then added to white opaque 96-well plates (Corning, 353296) and the Dual-Glo Luciferase Assay performed according to the manufacturer’s instructions (Promega, E2920). Firefly and *Renilla* luminescence were measured on a GloMax-Multi Detection System (Promega).

### RNA immunoprecipitation

RNA Immunoprecipitation was performed as described in [31] with some modifications. Briefly, an isogenic stable HEK293 cell line expressing the LMOD2 minigene containing the patient’s mutation (W398*) was transfected 2 days after plating with 100 nM of SBOs. The cells were washed 2X with ice-cold PBS on ice, collected in Polysome Lysis Buffer (PLB-100 mM KCl, 5 mM MgCl_2_ 10 mM HEPES-NaOH pH 7, 0.5% Nonident P-40, 1mM dithiothreitol, RNase Inhibitor-Murine [1U/μl; New England Biolabs, M0314L], 1X Halt Protease Inhibitor Cocktail [ThermoFisher Scientific, 78439]) with a cell lifter, pipetted to break up clumps, incubated on ice for 5 min and stored at -80°C. After thawing, the lysate was centrifuged at 16,000 × g for 15 min at 4°C. The supernatant was precleared with 20 μl of resuspended Protein A/G PLUS-Agarose (Santa Cruz Biotechnology, sc-2003) plus 2 μg of Normal Rabbit IgG (Cell Signalling, 2729) for 1 h at 4°C. After centrifugation, protein concentration of the supernatant was determined by BCA assay (ThermoFisher, 23225). To immobilize antibodies, 15 μg of rabbit polyclonal anti-eIF4AIII/EIF4A3 (ThermoFisher, A302-980A) or Normal Rabbit IgG was added to 20 μl of resuspended Protein A/G PLUS-Agarose in 100 μl of NT-2 buffer (50 mM Tris-HCl pH 7.4,150 mM NaCl, 1 mM MgCl_2_, 0.05% NP-40) and incubated with rotation for 1h at room temperature. Following 6 washes with NT-2 buffer, the antibody-bound beads were added to 2 mg of protein lysate in a total volume of 1 mL of PLB supplemented with 25 mM EDTA and incubated with rotation overnight at 4°C. After 6X washes with NT-2 buffer, a sample of the immunoprecipitate was processed for western blot analysis. The remaining immunoprecipitate was resuspended in 150 μL of Proteinase K buffer (1X NT-2 buffer + 1% sodium dodecyl sulfate) with 1.2 mg/ml of Proteinase K and incubated for 30 minutes at 55°C. RNA was then extracted using phenol-chloroform and quantified with a Qubit RNA HS Assay Kit (ThermoFisher, Q32852) and fluorometer. cDNA was synthesized using random hexamer primers from 100 ng of RNA as outlined above.

### Statistical analyses

All statistical analyses were performed using GraphPad Prism version 10.0 for macOS (GraphPad Software Inc.). Two groups were compared using Student’s t-tests (**Figs 1–4**). Multiple groups were compared using one-way ANOVAs with Tukey’s (**Fig 1**) or Dunnett’s (**Figs 4 and S1**) multiple comparison tests. p < 0.05 was considered significant. *P < .05, **P < .01, ***P < .001, ****P < .0001.

## Supporting information

S1 Fig Treatment with the steric-blocking oligonucleotides L2[-30], L2[-34] and L2[-38] recovers LMOD2[W398*] mutant protein and mature mRNA levels. **A**) Western blot analysis of LMOD2 proteins levels in FT-293 cells stably expressing *LMOD2* minigene (MG) constructs with or without the W398* mutation and treated with the indicated SBOs. Relative expression levels were determined following normalization to total protein levels assessed via Ponceau S staining. Data are means ± SD. n = 4; ***P <0.001, ****P <0.0001, Two-way ANOVA with Dunnett’s multiple comparison test. **B**) RT-qPCR analysis of *LMOD2* mature mRNA (left) and pre-mRNA (right) levels in FT-293 cells stably expressing *LMOD2* minigene constructs (MG) containing the W398* mutation treated with the indicated SBOs. Data are means ± SD. n = 9; *P <0.05, **P <0.01, ****P <0.0001, One-way ANOVA with Dunnett’s multiple comparison test.
(TIF)

S1 File Supplemental file containing sequence information. S1 File includes: site-directed mutagenesis and RT-qPCR primer sequences, steric-blocking oligonucleotide sequences, and *LMOD2* construct sequences.
(DOCX)

S2 File Numerical values underlying graphs in Figs 1–4 and S1. (XLSX)
