# Supplementary figures and images for: Human disease-causing mutations result in loss of leiomodin 2 through nonsense-mediated mRNA decay
Source: PLoS Genet. 2024 May 15;20(5):e1011279. doi: 10.1371/journal.pgen.1011279 (PMC11132695; doi:10.1371/journal.pgen.1011279)

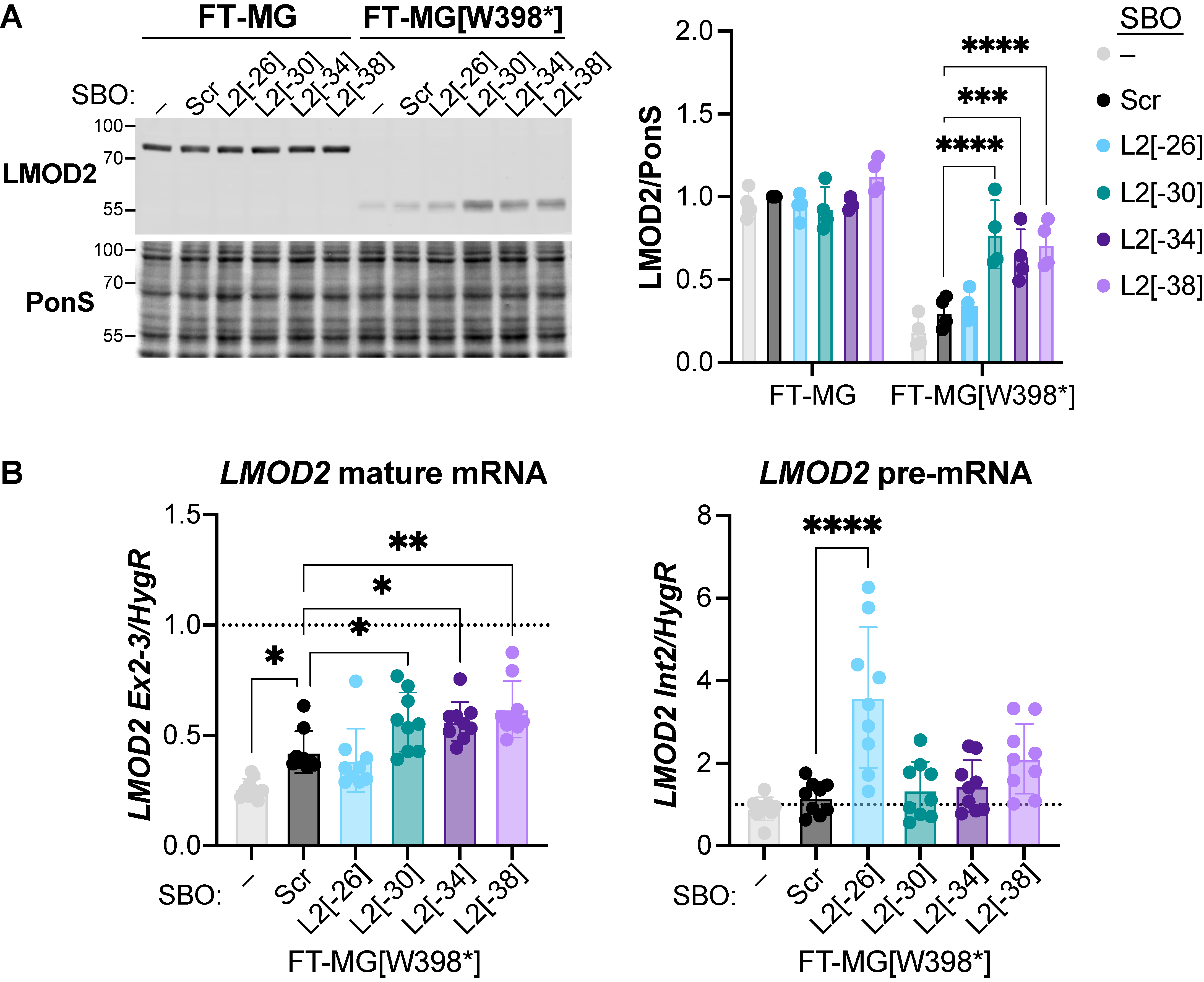

Supplement: S1 Fig — A) Western blot analysis of LMOD2 proteins levels in FT-293 cells stably expressing LMOD2 minigene (MG) constructs with or without the W398* mutation and treated with the indicated SBOs. Relative expression levels were determined following normalization to total protein levels assessed via Ponceau S staining. Data are means ± SD. n = 4; ***P <0.001, ****P <0.0001, Two-way ANOVA with Dunnett’s multiple comparison test. B) RT-qPCR analysis of LMOD2 mature mRNA (left) and pre-mRNA (right) levels in FT-293 cells stably expressing LMOD2 minigene constructs (MG) containing the W398* mutation treated with the indicated SBOs. Data are means ± SD. n = 9; *P <0.05, **P <0.01, ****P <0.0001, One-way ANOVA with Dunnett’s multiple comparison test. (TIF) [file pgen.1011279.s001.tif]
