# Supplementary material for: Human disease-causing mutations result in loss of leiomodin 2 through nonsense-mediated mRNA decay
Source: PLoS Genet. 2024 May 15;20(5):e1011279. doi: 10.1371/journal.pgen.1011279 (PMC11132695; doi:10.1371/journal.pgen.1011279)
Supplement: S1 File — includes: site-directed mutagenesis and RT-qPCR primer sequences, steric-blocking oligonucleotide sequences, and LMOD2 construct sequences. (DOCX) [file pgen.1011279.s002.docx]

**S1 File. Supplemental Sequence Information**

**Site-directed Mutagenesis Primer Sequences**

**hLMOD2 c.1193G>A, p.W398***

F: 5’-CTTATGTATCTCCCAGGCACTCACCCTAGTCATCC-3’

R: 5’-CCTGGGAGATACATAAGGTGAAGAGCTAGGTGTTCCT-3’

**hLMOD2 c.1243_1244 del, p.L415Vfs*108**

F: 5’-TCCAGACTGTGAGGAGCCGTCCTGTCTCCTGTGG-3’

R: 5’-CTCCTCACAGTCTGGACTTTTTTGGGGAGTTTTGGGGATG-3’

**hLMOD2 c.1537C>T, p.R513***

F: 5’-CAGTTCCTGACCTTCTACCCCACAGAGATCAGCTC-3’

R: 5’- GAAGGTCAGGAACTGTCTTCCATTTTCTTCTCTTGCAC-3’

**RT-qPCR Primer Sequences**

*LMOD2* Ex1-2 (spans intron 1):

F: 5′- GGACATTCAGCAGAGAGGCACT -3′

R: 5′- GATAAGCTCTTCTTCACTTTCCTC -3′

128-bp product; *E* = 1.834

*LMOD2* Ex2-3 (spans intron 2):

F: 5′- ACCCCACAGAGATCAGCTCA -3′

R: 5′- TATCGCAGGGCTTCTGGAAC -3′

98-bp product; *E* = 1.909

*LMOD2 Int2* (located within intron 2):

F: 5′- ACATTAGGAGCAGGCACACA -3′

R: 5′- AGGGAATTGGGACGTGTATGA -3′

119-bp product; *E* = 1.979

*RPL32*:

F: 5′- CACCAGTCAGACCGATATGTCAAAA -3′

R: 5′- TGTTGTCAATGCCTCTGGGTTT -3′

64-bp product; *E* = 1.912

Neomycin resistance gene:

F: 5′- GATGGATTGCACGCAGGTTC -3′

R: 5′- TCAGAGCAGCCGATTGTCTG -3′

86-bp product; *E* = 1.787

Hygromycin resistance gene:

F: 5′- ACAGCGGTCATTGACTGGAG -3′

R: 5′- TGCTGCTCCATACAAGCCAA -3′

101-bp product; *E* = 1.827

**Steric-blocking Oligonucleotide Sequences**

L2[-26]: 5’-TTTAGCTGTTTTATGCTGCTTC-3’

L2[-30]: 5’-TGTTTTATGCTGCTTCCCCG-3’

L2[-34]: 5’-TTATGCTGCTTCCCCGAATT-3’

L2[-38]: 5’-GCTGCTTCCCCGAATTGCTT-3’

Scrambled (Scr): 5’-TCTAACTACGCCTTGGTTTC-3’

***LMOD2* constructs**

**LMOD2 cDNA (CDS)**

ATGTCTACCTTTGGCTACCGAAGAGGACTCAGTAAATACGAATCCATCGACGAGGATGAACTCCTCGCCTCCCTGTCAGCCGAGGAGCTGAAGGAGCTAGAGAGAGAGTTGGAAGACATTGAACCTGACCGCAACCTTCCCGTGGGGCTAAGGCAAAAGAGCCTGACAGAGAAAACCCCCACAGGGACATTCAGCAGAGAGGCACTGATGGCCTATTGGGAAAAGGAGTCCCAAAAACTCTTGGAGAAGGAGAGGCTGGGGGAATGTGGAAAGGTTGCAGAAGACAAAGAGGAAAGTGAAGAAGAGCTTATCTTTACTGAAAGTAACAGTGAGGTTTCTGAGGAAGTGTATACAGAGGAGGAGGAGGAGGAGTCCCAGGAGGAAGAGGAGGAAGAAGACAGTGACGAAGAGGAAAGAACAATTGAAACTGCAAAAGGGATTAATGGAACTGTAAATTATGATAGTGTCAATTCTGACAACTCTAAGCCAAAGATATTTAAAAGTCAAATAGAGAACATAAATTTGACCAATGGCAGCAATGGGAGGAACACAGAGTCCCCAGCTGCCATTCACCCTTGTGGAAATCCTACAGTGATTGAGGACGCTTTGGACAAGATTAAAAGCAATGACCCTGACACCACAGAAGTCAATTTGAACAACATTGAGAACATCACAACACAGACCCTTACCCGCTTTGCTGAAGCCCTCAAGGACAACACTGTGGTGAAGACGTTCAGTCTGGCCAACACGCATGCCGACGACAGTGCAGCCATGGCCATTGCAGAGATGCTCAAAGTCAATGAGCACATCACCAACGTAAACGTCGAGTCCAACTTCATAACGGGAAAGGGGATCCTGGCCATCATGAGAGCTCTCCAGCACAACACGGTGCTCACGGAGCTGCGTTTCCATAACCAGAGGCACATCATGGGCAGCCAGGTGGAAATGGAGATTGTCAAGCTGCTGAAGGAGAACACGACGCTGCTGAGGCTGGGATACCATTTTGAACTCCCAGGACCAAGAATGAGCATGACGAGCATTTTGACAAGAAATATGGATAAACAGAGGCAAAAACGTTTGCAGGAGCAAAAACAGCAGGAGGGATACGATGGAGGACCCAATCTTAGGACCAAAGTCTGGCAAAGAGGAACACCTAGCTCTTCACCTTATGTATCTCCCAGGCACTCACCCTGGTCATCCCCAAAACTCCCCAAAAAAGTCCAGACTGTGAGGAGCCGTCCTCTGTCTCCTGTGGCCACACCTCCTCCTCCTCCCCCTCCTCCTCCTCCTCCCCCTCCTTCTTCCCAAAGGCTGCCACCACCTCCTCCTCCTCCCCCTCCTCCACTCCCAGAGAAAAAGCTCATTACCAGAAACATTGCAGAAGTCATCAAACAACAGGAGAGTGCCCAACGGGCATTACAAAATGGACAAAAAAAGAAAAAAGGGAAAAAGGTCAAGAAACAGCCAAACAGTATTCTAAAGGAAATAAAAAATTCTCTGAGGTCAGTGCAAGAGAAGAAAATGGAAGACAGTTCCCGACCTTCTACCCCACAGAGATCAGCTCATGAGAATCTCATGGAAGCAATTCGGGGAAGCAGCATAAAACAGCTAAAGCGGGTGGAAGTTCCAGAAGCCCTGCGATAA

**LMOD2 minigene (MG)**

ATGTCTACCTTTGGCTACCGAAGAGGACTCAGTAAATACGAATCCATCGACGAGGATGAACTCCTCGCCTCCCTGTCAGCCGAGGAGCTGAAGGAGCTAGAGAGAGAGTTGGAAGACATTGAACCTGACCGCAACCTTCCCGTGGGGCTAAGGCAAAAGAGCCTGACAGAGAAAACCCCCACAGGGACATTCAGCAGAGAGGCACTGATGGCCTATTGGGAAAAGGAGTCCCAAAAACTCTTGGAGAAGGAGAGGCTGGGGGAATGTGGAAAGGTTGCAGAAGACAAAGAGGAAAGTGAAGAAGAGCTTATCTTTACTGAAAGTAACAGTGAGGTTTCTGAGGAAGTGTATACAGAGGAGGAGGAGGAGGAGTCCCAGGAGGAAGAGGAGGAAGAAGACAGTGACGAAGAGGAAAGAACAATTGAAACTGCAAAAGGGATTAATGGAACTGTAAATTATGATAGTGTCAATTCTGACAACTCTAAGCCAAAGATATTTAAAAGTCAAATAGAGAACATAAATTTGACCAATGGCAGCAATGGGAGGAACACAGAGTCCCCAGCTGCCATTCACCCTTGTGGAAATCCTACAGTGATTGAGGACGCTTTGGACAAGATTAAAAGCAATGACCCTGACACCACAGAAGTCAATTTGAACAACATTGAGAACATCACAACACAGACCCTTACCCGCTTTGCTGAAGCCCTCAAGGACAACACTGTGGTGAAGACGTTCAGTCTGGCCAACACGCATGCCGACGACAGTGCAGCCATGGCCATTGCAGAGATGCTCAAAGTCAATGAGCACATCACCAACGTAAACGTCGAGTCCAACTTCATAACGGGAAAGGGGATCCTGGCCATCATGAGAGCTCTCCAGCACAACACGGTGCTCACGGAGCTGCGTTTCCATAACCAGAGGCACATCATGGGCAGCCAGGTGGAAATGGAGATTGTCAAGCTGCTGAAGGAGAACACGACGCTGCTGAGGCTGGGATACCATTTTGAACTCCCAGGACCAAGAATGAGCATGACGAGCATTTTGACAAGAAATATGGATAAACAGAGGCAAAAACGTTTGCAGGAGCAAAAACAGCAGGAGGGATACGATGGAGGACCCAATCTTAGGACCAAAGTCTGGCAAAGAGGAACACCTAGCTCTTCACCTTATGTATCTCCCAGGCACTCACCCTGGTCATCCCCAAAACTCCCCAAAAAAGTCCAGACTGTGAGGAGCCGTCCTCTGTCTCCTGTGGCCACACCTCCTCCTCCTCCCCCTCCTCCTCCTCCTCCCCCTCCTTCTTCCCAAAGGCTGCCACCACCTCCTCCTCCTCCCCCTCCTCCACTCCCAGAGAAAAAGCTCATTACCAGAAACATTGCAGAAGTCATCAAACAACAGGAGAGTGCCCAACGGGCATTACAAAATGGACAAAAAAAGAAAAAAGGGAAAAAGGTCAAGAAACAGCCAAACAGTATTCTAAAGGAAATAAAAAATTCTCTGAGGTCAGTGCAAGAGAAGAAAATGGAAGACAGTTCCCGACCTTCTACCCCACAGAGATCAGCTCATGAGAATCTCATGGAAGCAATTCGGGGAAGCAGCATAAAACAGCTAAAGCGGGTAAGTAACCAGAGAACAGACATAGGGGCACAGATAAAGTAAATGAGTTGTCCTCCATTGCATGGTGGTACCAAAGTCACCTCTCACAATACTTATCAATACTTTCAATATTTTAGTATGCGAGAGCAAACACACCAAGTTTGAAACATTAGGAGCAGGCACACAAGTGAGCACATTTCTATTTGAGAGGAACGCCTGGGCCGCTTTCCCAGCCTCTCAATCATATAAGGGCAAGGACCATTTTCATACACGTCCCAATTCCCTTAAGAAGAAAACCAGGGAATATAATCTAAATTCCAAAAGGATTCACCACTTGTCAAAAATTTTACCACAGAGTTGTGACTGATTCGATACCTAATTTATAACATAAAATTTATAATGTGGTAAAAAAATTACCATGCAGCAATAATCAACCTGAGTTCTTCTCAGAGGATACTGCTAGAGACATATCCTACAGATATTTTTCACTTATCATGTGTGTTGTTTCATTGAGAGAAAATCCGCTATTTTTGTAGGTGGAAGTTCCAGAAGCCCTGCGATAAAAACATGATCTTTAGAAGAGGATGCAGAACTGTTCAGTGGTATTACATGAAATGCATTGTGAGATGTTTCTAAAATACCTTCTTCAATTCAAAATGATCCCTGACTTTAAAAATAATCTCACCCATTAATTCCAAAGAGAATCTTAAGAAACAATCAGCATGTTTCTTCTGTAAATATGAAAATAAATTTCTTTTTTATGTCGTGAGATTTGTATTGGCAAGAAGCAGTTAATTTAAAGATGCTCTTCCTATCTGTGGATGTGTTGGTAACTCCGAGTTGTAATGAGTTCATGAAATGTGCTGTTATTTTTGTAATCTCAATAAATGTGGATTGAAGTTTTTTCCCTTTTTTTAAAGCCAAACTAATATTTTTCTGTGACTTGATACATCTGTCAGATTTTTGTAATCTCGATAAATGTGTATTGAAGTTTTTTCCCTTTTTTTAAAAAGCCAAACTAATATTTTTCTGTGAGTTAATACATCTGTCAGGTGTGTATGTAACATTACTGGACATTAAAAAAAAATATTACATTCTCACCCAAAAAGGGTTTGGGTCCTAAGCATTTGCCTTTCTTTGTTTCCTCTTGTTCAAGAAAATCTGATTAGATCTCTTTCTAAAGGACTGCAAGCAATAATTTTTTTTTATATTTTATTTATTTATTTATTTTTTTGAGACAAGTCTTGCTCTGTTACCCA

**LMOD2 minigene (MG) + Beta-globin intron 2**

ATGTCTACCTTTGGCTACCGAAGAGGACTCAGTAAATACGAATCCATCGACGAGGATGAACTCCTCGCCTCCCTGTCAGCCGAGGAGCTGAAGGAGCTAGAGAGAGAGTTGGAAGACATTGAACCTGACCGCAACCTTCCCGTGGGGCTAAGGCAAAAGAGCCTGACAGAGAAAACCCCCACAGGGACATTCAGCAGAGAGGCACTGATGGCCTATTGGGAAAAGGAGTCCCAAAAACTCTTGGAGAAGGAGAGGCTGGGGGAATGTGGAAAGGTTGCAGAAGACAAAGAGGAAAGTGAAGAAGAGCTTATCTTTACTGAAAGTAACAGTGAGGTTTCTGAGGAAGTGTATACAGAGGAGGAGGAGGAGGAGTCCCAGGAGGAAGAGGAGGAAGAAGACAGTGACGAAGAGGAAAGAACAATTGAAACTGCAAAAGGGATTAATGGAACTGTAAATTATGATAGTGTCAATTCTGACAACTCTAAGCCAAAGATATTTAAAAGTCAAATAGAGAACATAAATTTGACCAATGGCAGCAATGGGAGGAACACAGAGTCCCCAGCTGCCATTCACCCTTGTGGAAATCCTACAGTGATTGAGGACGCTTTGGACAAGATTAAAAGCAATGACCCTGACACCACAGAAGTCAATTTGAACAACATTGAGAACATCACAACACAGACCCTTACCCGCTTTGCTGAAGCCCTCAAGGACAACACTGTGGTGAAGACGTTCAGTCTGGCCAACACGCATGCCGACGACAGTGCAGCCATGGCCATTGCAGAGATGCTCAAAGTCAATGAGCACATCACCAACGTAAACGTCGAGTCCAACTTCATAACGGGAAAGGGGATCCTGGCCATCATGAGAGCTCTCCAGCACAACACGGTGCTCACGGAGCTGCGTTTCCATAACCAGAGGCACATCATGGGCAGCCAGGTGGAAATGGAGATTGTCAAGCTGCTGAAGGAGAACACGACGCTGCTGAGGCTGGGATACCATTTTGAACTCCCAGGACCAAGAATGAGCATGACGAGCATTTTGACAAGAAATATGGATAAACAGAGGCAAAAACGTTTGCAGGAGCAAAAACAGCAGGAGGGATACGATGGAGGACCCAATCTTAGGACCAAAGTCTGGCAAAGAGGAACACCTAGCTCTTCACCTTATGTATCTCCCAGGCACTCACCCTGGTCATCCCCAAAACTCCCCAAAAAAGTCCAGACTGTGAGGAGCCGTCCTCTGTCTCCTGTGGCCACACCTCCTCCTCCTCCCCCTCCTCCTCCTCCTCCCCCTCCTTCTTCCCAAAGGCTGCCACCACCTCCTCCTCCTCCCCCTCCTCCACTCCCAGAGAAAAAGCTCATTACCAGAAACATTGCAGAAGTCATCAAACAACAGGAGAGTGCCCAACGGGCATTACAAAATGGACAAAAAAAGAAAAAAGGGAAAAAGGTCAAGAAACAGCCAAACAGTATTCTAAAGGAAATAAAAAATTCTCTGAGGTCAGTGCAAGAGAAGAAAATGGAAGACAGTTCCCGACCTTCTACCCCACAGAGATCAGCTCATGAGAATCTCATGGAAGCAATTCGGGGAAGCAGCATAAAACAGCTAAAGCGGGTGAGTCTATGGGACGCTTGATGTTTTCTTTCCCCTTCTTTTCTATGGTTAAGTTCATGTCATAGGAAGGGGATAAGTAACAGGGTACAGTTTAGAATGGGAAACAGACGAATGATTGCATCAGTGTGGAAGTCTCAGGATCGTTTTAGTTTCTTTTATTTGCTGTTCATAACAATTGTTTTCTTTTGTTTAATTCTTGCTTTCTTTTTTTTTCTTCTCCGCAATTTTTACTATTATACTTAATGCCTTAACATTGTGTATAACAAAAGGAAATATCTCTGAGATACATTAAGTAACTTAAAAAAAAACTTTACACAGTCTGCCTAGTACATTACTATTTGGAATATATGTGTGCTTATTTGCATATTCATAATCTCCCTACTTTATTTTCTTTTATTTTTAATTGATACATAATCATTATACATATTTATGGGTTAAAGTGTAATGTTTTAATATGTGTACACATATTGACCAAATCAGGGTAATTTTGCATTTGTAATTTTAAAAAATGCTTTCTTCTTTTAATATACTTTTTTGTTTATCTTATTTCTAATACTTTCCCTAATCTCTTTCTTTCAGGGCAATAATGATACAATGTATCATGCCTCTTTGCACCATTCTAAAGAATAACAGTGATAATTTCTGGGTTAAGGCAATAGCAATATCTCTGCATATAAATATTTCTGCATATAAATTGTAACTGATGTAAGAGGTTTCATATTGCTAATAGCAGCTACAATCCAGCTACCATTCTGCTTTTATTTTATGGTTGGGATAAGGCTGGATTATTCTGAGTCCAAGCTAGGCCCTTTTGCTAATCATGTTCATACCTCTTATCTTCCTCCCACAGGTGGAAGTTCCAGAAGCCCTGCGATAAAAACATGATCTTTAGAAGAGGATGCAGAACTGTTCAGTGGTATTACATGAAATGCATTGTGAGATGTTTCTAAAATACCTTCTTCAATTCAAAATGATCCCTGACTTTAAAAATAATCTCACCCATTAATTCCAAAGAGAATCTTAAGAAACAATCAGCATGTTTCTTCTGTAAATATGAAAATAAATTTCTTTTTTATGTCGTGAGATTTGTATTGGCAAGAAGCAGTTAATTTAAAGATGCTCTTCCTATCTGTGGATGTGTTGGTAACTCCGAGTTGTAATGAGTTCATGAAATGTGCTGTTATTTTTGTAATCTCAATAAATGTGGATTGAAGTTTTTTCCCTTTTTTTAAAGCCAAACTAATATTTTTCTGTGACTTGATACATCTGTCAGATTTTTGTAATCTCGATAAATGTGTATTGAAGTTTTTTCCCTTTTTTTAAAAAGCCAAACTAATATTTTTCTGTGAGTTAATACATCTGTCAGGTGTGTATGTAACATTACTGGACATTAAAAAAAAATATTACATTCTCACCCAAAAAGGGTTTGGGTCCTAAGCATTTGCCTTTCTTTGTTTCCTCTTGTTCAAGAAAATCTGATTAGATCTCTTTCTAAAGGACTGCAAGCAATAATTTTTTTTTATATTTTATTTATTTATTTATTTTTTTGAGACAAGTCTTGCTCTGTTACCCA

**LMOD2 gene (G)**

ATGTCTACCTTTGGCTACCGAAGAGGACTCAGTAAATACGAATCCATCGACGAGGATGAACTCCTCGCCTCCCTGTCAGCCGAGGAGCTGAAGGAGCTAGAGAGAGAGTTGGAAGACATTGAACCTGACCGCAACCTTCCCGTGGGGCTAAGGCAAAAGAGCCTGACAGAGAAAACCCCCACAGGGACATTCAGCAGAGAGGCACTGATGGCCTATTGGGAAAAGGAGTCCCAAAAACTCTTGGAGAAGGAGAGGCTGGGGGAATGTGGAAAGGTAGGCTCTCGGGACTTTTCCTTGGCTAACCCCACCTCCCCATCACCCCATCCCAAACCCAGACACTTGTTTTCCCTAACTTCTCAATCCTCATCACTTGAATTTTCCTATAACCCATTTATACTTGCTACTAAATCATTTTTCAGGCTGAAATAATCATGTAAATGCTATGGCCACCCAATGGATCATAATATGTAAAATTCTTAATTGGGGCTCTTTGATTGATTACAAATAGAAAGCTCAAGAGGAAAATAATTCACAATCAGTGTATGGGATTCAATATTTTGCCAAACATAAGTGTTGTTTGAGCTATATGTTGGTAATGTGGCATAATATTTGTTCCCAAGATACATGCATACGTATAAATGTATACTTTTTTTTCAGTTAAGTACATTTATTTTCTCCTATAAAGACAAAAGTGGGAACGCTGGTTGCTTTGACTGTGATAGTAGCTATGGTGAAATAGTTCTTTTCCAATACCAAGAAGTGTGGGGTGGTGGGAAGTACATGACACTGAGAGTCACAGGGCCTTGTTATGAGTCCCTGCTCTGTTCCCACATCTTTAGACCTTGAACAATCACTTTTACAGGGTTCTCTTTCCCTCTGGACATTGAGGGAGTTAGAGACAGTGTTTCTTTTGCGAATCAAGAACCCCTTTGAGATTTTGATAAGTTATGAACCTTCTTAAGAAAATAGCCCTGGGCTTGTGCAAAATACACCAACTTTTGCATAGAATTTCAAAATGATAACACATCCTGAAACCCATCTGAGGACTCAGGGGCAGCACAGCTGTATAGATGATCTCTTCGATTTCTGTCTCCTTTACGATGGTGATCCTGAGGTTAGGAAATGTGCTTATATCAGAGCTCAGGCCTATAAAGCTCTTGTGTACTGCCCAGTAAACAGTTATTTTGAATTTGCCAAATGAGTCAATACCAGAAGGAAGTCCAAAAGACTATTATTCTCTGGGAAAACAGTAATGACAATAAAGTGTCCATATAACAAAAAAAAAATTCTTCCGGAATTATGGAAGAACATAGGTCAGGAGAAACAGTTTAAAACCCTTGCACTCTGTTGGAATCATCTAAAATCAAAATGGCCTAAAGCCAATGGCAAGACTTCCACAGTACTAATCTAGGGCTGTTAGATGATCTTGATGATAGGGTTATCTTGGCCACAATTGCAGCAAGATATGTGTGAGTTTTGTCCACGTCAATCATTGCAGGAAGGGCCACTGGGATCTGGCCAGGGTCTGCCAAGGCCACAGTACTAATCAGCTAGAAGGGGTGTGTTAGAAGGAAGGACACATACAAGTCAGTACTTGGGCAGGTCAATTCCTCCACAACCATTGTTTTAAGAGGGATTTTTTTTTCTTTGATGTGATAATGAGCATCAAAATAACAATGACATTATCATCACAATTATTTAAAACTCTCTTTAATAAAATAAAACTGGCTGGGTATGCTGGCTCACGCCTATAATTCCCACACTTTTTTGGGAGGCTGAGGCAGGAGAATCACTTGAGCCTAGGAGTTCAAATCCAGCTCTGGCAATATAGTGAGATCTCATCTCTACAAAAAAAAAAAAAAAAAAAAAAAATTAGCTGGGTTTAGTGGAACATTAGCAGGTGGTAGTCCCAGCTGCTCAGGAGGCTGAGGTGGGAGGTTGGCTTCAGCATGGGAGGTTGAGGCTGCAGTGAGCTATGGTTGTGCCACTGCACTCAGCCTGAGCCACAAACCAAAACCTTGTCTCATTAAAAAAAAAAAAAAAAAAAAAGAATAAAACTGTCTCAGAATATCCAGGCTGTACAACCAATGTATCAATAATCCAACAAAGGCTTTTTAATGTCACAATGACATGATAGTGGTGGGAACCAAAGAGCAAAACCTATCGTCAGCCGGACGAACCCACCTGATCCTGCCAAAGGTTTTGCTGATCCCTGCCAATGGCTTATCAGCTCAATTAGAGAGGCTGCTGTGTTGATGGACTTCGGTTCCTTTCTAGGCTATTAACTTTTTCTCCCAGTACTTTCCCTGCTGGCCAGCTGTCTGTAGTTGCCAGGCATGATGAGCAGGAACTTGCTGATGGAGGGTGCAAATCCAATTCCCACAACAGGGAAAATCTTCGAGCAGCTGTTGGGTGGCTGGGGGAGCAAGGTAGTTGCGTAAGTGAAGAGCCATAAAGGAAGGACCCTGAGAAGGAGCGGAGGGTCTTGATTGATTATCAGGCGGCTGGCTCTGTAAAGGCTTATAAAAAGCAGATGCAACACCTGTTGAATGCCAGCCTTTCTGCCCACTTGGGCTTACACAAACTTATGCCGCATTGGGGCTCCCAGGGACCCTAGAATCCCAATGGTCTAGCTCCCCTATTGAGCAGTGGTTAAATCTCAGCACAGGTGCCAATGAGTTTCTCCCATTATCTCACTTCATTTTTAACTTTCTGCACTTGAGAACTGAGACAAGGCAAGGTTAAGAAACTCATTAGAGGTTAAGAGCTTGTACATAGAAGTGAAAATTAAAGAACATTTCAAAATTGTGGGTGCCCGGTCAGCAGCATCAGCATTACCTGGGATCTTCTTGGAACTGCAAATTATAGAGTTCCCGTCAATCTGATGAATGATAAACTCTGGGGATGGGGCCCAACAATTGGTGCTCTCACAGGTGATTATGACACACAGTAAAACTTAAGAACCGCTCAACTACAGTTACCTCCTGAATAACATGGGGGTCAGGTGAACCAACTCCTGCCCCTGCAGCATCCAAAATTCATGTATAACTTTTGACTCCCCCAAACCTTAACTACTAATAGCCTACTGTGGACAGAAGCCTTACCAATAACATAAACGGTCAATTAACACATACTTTGAATGTGATATGTACATACATAATAGGGAGGTGATCAGGAGAAACAACCGGCCACTCTTCTCATTACCTGATTTGTTTTCTAAGTTTACTAACATTCCCCTTCTCCTTGTCCAGTCCCTTGCCATCTCATCTCCCACCTCCTTATTCTATACCCTCCTTCCCACCTCCATTCTCCAAAGAATCAGGGTTAATGCCAAAGCAGTGGCATATAGGAAATCTGTAATAAAACAGGCCCCCTAGGGACAGAGTATTTTATAAAGTAAGCGAAGTCTGTTTTTTGCATCCTCTCACCTTGCCTCTGTAGACACCTTTTCCCCTTCTGTTCTTTCTCCAGATGTCCCCCACCTAGGGTGACTAATCATCTCAGATTGTCCGGAACTATCTAGATTTCATGACTAAAATCCTGGGAAACCCTTCACACTCAGGCAAACTGGGAGGGTTTATCACCTTTCTCCTGTGTGTAGTCCTGACTCTCAGCTGCTGTTAACCAATGTCAACTTAGAGCCATTCAGTAAGCAAATAATTATTGAGCTCCTACTACATGCTAAGCACTGTGCTAGGTGAGCAAAGCAGACATGGTCCACTGCTCTCAACAGCTAACAGTCTGCTGGGAAAAACAAGTGACCAAAATAAAGTGCGACAGGTACTTTATTGGGGGTGGGTACAATGGTATGGGAATGCGTCTGAGGGATATTTGGAGAGGATGTCAAAGAAGGCTTTCTGGGAGATCTATCATTTAGATGCTATATGACAATTCACAGTTTCAGATATTATCTGTCCAGGTACATTATAAGGGGAAAACAGATCTTGCAGATAGAGCCAGAGGGTCTTTAAAAAACCAAAGGGAGTAAAATGTAATGGTGTCATACAAGGCCGTATACCTTGGATTTTGAGACAGGCCCCTTTCCTCCAAATACTGAAACAGCTAAAAATACATACAAACACATGAACACACACAGCTTTACAGGGACTAGAATTATAAGGAATTTGGAGTGGAATGGGGTGAGGAGAAGTCAGCCCACAATTCTTTCCTTCCTCGGGTTAATCCATTTGTTTCACTGCTGGACAGGGAAGGCTTCCTCTCTTCCCTCACGTATGCCTGGAGACTTGGCTAGAGCCACACTCTTTTTCTTATGTTGTTGCATTGTGACTTGCAGCCAGCTGCCACCAAACATTTCTCTCTCTTTCTCTCTCTCTCTCTCTCTCCCTCTCTCTCTCTCTCTCACACACACACACACACACAGAGACACACAGAGTATCTCTCTCTCTCCCTCCCTGCTCCTGAAGAAGTGAATAAATTAAGACTGGAAAAGTCATCCCAGGAGTGGCTGACCCCTTCCCCAGGCCATCACTCGGTGCCTGCCACTCCAAACAGTGGATAACTGGAGGCTATTTTGTGATAGCAACTGGGCAAACGAACCTGGAGACAAGGGCAGTGTTTTCCAAAGCTAGATCTACATGCTTCAGGCAGCGGGCGCCCTCGAGGTCAAAAAAAAAAAAGTTGCCAATATAGAGAGAAGAATCAGGGCCTGGAGGACAGGGCAGGGTCCCAGGGCTGTAGAGAAGATAAAGAGAAAGGACTAAAAAGACAGCAGGAGAAAAAGATTAGACTCTGTCAGCAATTTCTAGACCTTTTTTTTTTTTAATCAGAAAAGATAGCAAGAAAAAGAGAAACAAGGGGTCACTTGAGCGTCTTTGTACAGCAAATCTTGTAAACAGGAAAGACAAAGATAGAATGAAATTGTGTTTGCCTGAAAGCCTTTCATAATTGATACATGCTAAATAATTTAAGACCTTCAGGCTGGGCAATGGCTTACTTCAAGATTTTCTGCTGTTCTCTGCTGTTCCATCCACTTTACTGATTAGAATCTAGTGGATGCCAAGGGTGATTATCTTTCCCACCTGGTCTTCTACAGTCTGTCTCATTTAACCCTGGACTAGTTGGAGGTTTCAAGTTGATTTTCCCAAAAGGGTGACCTGAATGTTTTCTCTGCATCGAACTGGATTTTGACCTCCTGCTGTCCTGAGCAGCAGCCCTGCCTTTAGGCTTCCCTTGCCCAGATAAGGGACAGCTACAGAGGCAGCTCTCAGGTGATTCAGAGAGTCTTCTGTGGTAGAGCCCTGTGCCGGGCTCTACCGTAGAAGCAGATAAATAAATGCTGGAGGAGCAAATGACAGCTCTGCTTCTGTCTACACTGAGGCAAGGCCTGGCAGAGAGTGGTTGGCTTCTGTCACATCTGCTATGGATGGAAAATTTGGGAATTTTTGAATTAAAAAGGGAAACTCATTAACATATTGTATATTTTTGTTGTCATTTGATTGCTTGAGATGGAGAAATAAGAAAGAACAGAGTGATTCCAGAAGGGAGGGTTGAATTTGCTACATTGTAAGTAAATGGAGAGTAAACCAGTAAGAGTCGGTCCTCAGGAGGTGAACAAATTTATCAAGGGTTTTCCAAAGCTGAAGTTCTGATTTATTTGTTCAAATTAGCAAAATGATTATATATTTTTATTCAATAAGTTGAATTCTGGATATTCTGCAGAAAACAAGAGAAATGTTTGTAGAAAAAGCTATTGCTATTTATTGCAATAAAATGTCAGCCTTGGTCATGAGAACTAGACAGAATGGAAGCAGAGGATTATTAACTTTTTTTATTATAAACTTATGTCACTTGCCATGTGCTACTTTTGCAAGTTAAAAAATGTACTTAAAAATGGTATCATTTTTAAGAAGCTTAATGATGATATCATACTCTTTAGGTTGCAGAAGACAAAGAGGAAAGTGAAGAAGAGCTTATCTTTACTGAAAGTAACAGTGAGGTTTCTGAGGAAGTGTATACAGAGGAGGAGGAGGAGGAGTCCCAGGAGGAAGAGGAGGAAGAAGACAGTGACGAAGAGGAAAGAACAATTGAAACTGCAAAAGGGATTAATGGAACTGTAAATTATGATAGTGTCAATTCTGACAACTCTAAGCCAAAGATATTTAAAAGTCAAATAGAGAACATAAATTTGACCAATGGCAGCAATGGGAGGAACACAGAGTCCCCAGCTGCCATTCACCCTTGTGGAAATCCTACAGTGATTGAGGACGCTTTGGACAAGATTAAAAGCAATGACCCTGACACCACAGAAGTCAATTTGAACAACATTGAGAACATCACAACACAGACCCTTACCCGCTTTGCTGAAGCCCTCAAGGACAACACTGTGGTGAAGACGTTCAGTCTGGCCAACACGCATGCCGACGACAGTGCAGCCATGGCCATTGCAGAGATGCTCAAAGTCAATGAGCACATCACCAACGTAAACGTCGAGTCCAACTTCATAACGGGAAAGGGGATCCTGGCCATCATGAGAGCTCTCCAGCACAACACGGTGCTCACGGAGCTGCGTTTCCATAACCAGAGGCACATCATGGGCAGCCAGGTGGAAATGGAGATTGTCAAGCTGCTGAAGGAGAACACGACGCTGCTGAGGCTGGGATACCATTTTGAACTCCCAGGACCAAGAATGAGCATGACGAGCATTTTGACAAGAAATATGGATAAACAGAGGCAAAAACGTTTGCAGGAGCAAAAACAGCAGGAGGGATACGATGGAGGACCCAATCTTAGGACCAAAGTCTGGCAAAGAGGAACACCTAGCTCTTCACCTTATGTATCTCCCAGGCACTCACCCTGGTCATCCCCAAAACTCCCCAAAAAAGTCCAGACTGTGAGGAGCCGTCCTCTGTCTCCTGTGGCCACACCTCCTCCTCCTCCCCCTCCTCCTCCTCCTCCCCCTCCTTCTTCCCAAAGGCTGCCACCACCTCCTCCTCCTCCCCCTCCTCCACTCCCAGAGAAAAAGCTCATTACCAGAAACATTGCAGAAGTCATCAAACAACAGGAGAGTGCCCAACGGGCATTACAAAATGGACAAAAAAAGAAAAAAGGGAAAAAGGTCAAGAAACAGCCAAACAGTATTCTAAAGGAAATAAAAAATTCTCTGAGGTCAGTGCAAGAGAAGAAAATGGAAGACAGTTCCCGACCTTCTACCCCACAGAGATCAGCTCATGAGAATCTCATGGAAGCAATTCGGGGAAGCAGCATAAAACAGCTAAAGCGGGTAAGTAACCAGAGAACAGACATAGGGGCACAGATAAAGTAAATGAGTTGTCCTCCATTGCATGGTGGTACCAAAGTCACCTCTCACAATACTTATCAATACTTTCAATATTTTAGTATGCGAGAGCAAACACACCAAGTTTGAAACATTAGGAGCAGGCACACAAGTGAGCACATTTCTATTTGAGAGGAACGCCTGGGCCGCTTTCCCAGCCTCTCAATCATATAAGGGCAAGGACCATTTTCATACACGTCCCAATTCCCTTAAGAAGAAAACCAGGGAATATAATCTAAATTCCAAAAGGATTCACCACTTGTCAAAAATTTTACCACAGAGTTGTGACTGATTCGATACCTAATTTATAACATAAAATTTATAATGTGGTAAAAAAATTACCATGCAGCAATAATCAACCTGAGTTCTTCTCAGAGGATACTGCTAGAGACATATCCTACAGATATTTTTCACTTATCATGTGTGTTGTTTCATTGAGAGAAAATCCGCTATTTTTGTAGGTGGAAGTTCCAGAAGCCCTGCGATAAAAACATGATCTTTAGAAGAGGATGCAGAACTGTTCAGTGGTATTACATGAAATGCATTGTGAGATGTTTCTAAAATACCTTCTTCAATTCAAAATGATCCCTGACTTTAAAAATAATCTCACCCATTAATTCCAAAGAGAATCTTAAGAAACAATCAGCATGTTTCTTCTGTAAATATGAAAATAAATTTCTTTTTTATGTCGTGAGATTTGTATTGGCAAGAAGCAGTTAATTTAAAGATGCTCTTCCTATCTGTGGATGTGTTGGTAACTCCGAGTTGTAATGAGTTCATGAAATGTGCTGTTATTTTTGTAATCTCAATAAATGTGGATTGAAGTTTTTTCCCTTTTTTTAAAGCCAAACTAATATTTTTCTGTGACTTGATACATCTGTCAGATTTTTGTAATCTCGATAAATGTGTATTGAAGTTTTTTCCCTTTTTTTAAAAAGCCAAACTAATATTTTTCTGTGAGTTAATACATCTGTCAGGTGTGTATGTAACATTACTGGACATTAAAAAAAAATATTACATTCTCACCCAAAAAGGGTTTGGGTCCTAAGCATTTGCCTTTCTTTGTTTCCTCTTGTTCAAGAAAATCTGATTAGATCTCTTTCTAAAGGACTGCAAGCAATAATTTTTTTTTATATTTTATTTATTTATTTATTTTTTTGAGACAAGTCTTGCTCTGTTACCCA
